# Supplementary material for: Multi‐dataset identification of innovative feature genes and molecular mechanisms in keratoconus
Source: J Cell Mol Med. 2024 Sep 19;28(18):e70079. doi: 10.1111/jcmm.70079 (PMC11412914; doi:10.1111/jcmm.70079)
Supplement: Supplementary file 2 — Material S2. [file JCMM-28-e70079-s002.zip › 7.GSEA analysis/3.DBNDD1/KEGG_BIOSYNTHESIS_OF_UNSATURATED_FATTY_ACIDS.html]

Details for gene set KEGG\_BIOSYNTHESIS\_OF\_UNSATURATED\_FATTY\_ACIDS[GSEA]

|  || Dataset | DBNDD1.group.cls#h\_versus\_l |
| Phenotype | group.cls#h\_versus\_l |
| Upregulated in class | l |
| GeneSet | KEGG\_BIOSYNTHESIS\_OF\_UNSATURATED\_FATTY\_ACIDS |
| Enrichment Score (ES) | -0.36688647 |
| Normalized Enrichment Score (NES) | -1.2776681 |
| Nominal p-value | 0.14629258 |
| FDR q-value | 0.69032305 |
| FWER p-Value | 0.971 |
Table: GSEA Results Summary

  

Fig 1: Enrichment plot: KEGG\_BIOSYNTHESIS\_OF\_UNSATURATED\_FATTY\_ACIDS      
 Profile of the Running ES Score & Positions of GeneSet Members on the Rank Ordered List

  

| SYMBOL | TITLE | RANK IN GENE LIST | RANK METRIC SCORE | RUNNING ES | CORE ENRICHMENT || 1 | SCD5 | na | 6018 | 0.150 | -0.0800 | No |
| 2 | ACAA1 | na | 9690 | 0.076 | -0.1386 | No |
| 3 | HSD17B12 | na | 9708 | 0.075 | -0.0921 | No |
| 4 | FADS2 | na | 9812 | 0.074 | -0.0490 | No |
| 5 | ACOX1 | na | 10838 | 0.060 | -0.0412 | No |
| 6 | PECR | na | 11380 | 0.054 | -0.0234 | No |
| 7 | ACOX3 | na | 11488 | 0.052 | 0.0061 | No |
| 8 | ACOT7 | na | 11577 | 0.051 | 0.0356 | No |
| 9 | ELOVL6 | na | 12018 | 0.047 | 0.0521 | No |
| 10 | TECR | na | 13750 | 0.032 | 0.0222 | No |
| 11 | ACOT4 | na | 14896 | 0.024 | 0.0039 | No |
| 12 | SCD | na | 16749 | 0.011 | -0.0426 | No |
| 13 | HADHA | na | 17010 | 0.009 | -0.0443 | No |
| 14 | BAAT | na | 18325 | 0.001 | -0.0818 | No |
| 15 | ACOT1 | na | 18665 | -0.002 | -0.0903 | No |
| 16 | FADS1 | na | 28265 | -0.137 | -0.2814 | Yes |
| 17 | ELOVL2 | na | 28824 | -0.152 | -0.2028 | Yes |
| 18 | YOD1 | na | 30274 | -0.197 | -0.1220 | Yes |
| 19 | ELOVL5 | na | 33787 | -0.402 | 0.0271 | Yes |
Table: GSEA details [plain text format]

  

Fig 2: KEGG\_BIOSYNTHESIS\_OF\_UNSATURATED\_FATTY\_ACIDS      
 Blue-Pink O' Gram in the Space of the Analyzed GeneSet

  

Fig 3: KEGG\_BIOSYNTHESIS\_OF\_UNSATURATED\_FATTY\_ACIDS: Random ES distribution      
 Gene set null distribution of ES for **KEGG\_BIOSYNTHESIS\_OF\_UNSATURATED\_FATTY\_ACIDS**
